# The Impact of Advice Seekers’ Need Salience and Doctors’ Communication Style on Attitude and Decision Making: A Web-Based Mammography Consultation Role Play

**DOI:** 10.2196/cancer.4279

**Published:** 2015-09-08

**Authors:** Tim Fissler, Martina Bientzle, Ulrike Cress, Joachim Kimmerle

**Affiliations:** ^1^ Leibniz-Institut fuer Wissensmedien, Knowledge Media Research Center Knowledge Construction Lab Tuebingen Germany; ^2^ Department of Psychology University of Tuebingen Tuebingen Germany

**Keywords:** communication style, needs, need salience, attitude, decision-making, mammography screening, online consultation

## Abstract

**Background:**

Patients and advice seekers come to a medical consultation with typical needs, and physicians require adequate communication skills in order to address those needs effectively. It is largely unclear, however, to what extent advice seekers’ attitudes toward a medical procedure or their resulting decisions are influenced by a physician’s communication that ignores or explicitly takes these needs into account.

**Objective:**

This experimental study tested how advice seekers’ salient needs and doctor’s communication styles influenced advice seekers’ attitudes toward mammography screening and their decision whether or not to participate in this procedure.

**Methods:**

One hundred women (age range 20-47 years, mean 25.22, SD 4.71) participated in an interactive role play of an online consultation. During the consultation, a fictitious, program-controlled physician provided information about advantages and disadvantages of mammography screening. The physician either merely communicated factual medical information or made additional comments using a communication style oriented toward advice seekers’ typical needs for clarity and well-being. Orthogonal to this experimental treatment, participants’ personal needs for clarity and for well-being were either made salient before or after the consultation with a needs questionnaire. We also measured all participants’ attitudes toward mammography screening and their hypothetical decisions whether or not to participate before and after the experiment.

**Results:**

As assumed, the participants expressed strong needs for clarity (mean 4.57, SD 0.42) and for well-being (mean 4.21, SD 0.54) on 5-point Likert scales. Making these needs salient or not revealed significant interaction effects with the physician’s communication style regarding participants’ attitude change (*F*_1,92_=7.23, *P*=.009, η^2^=.073) and decision making (*F*_1,92_=4.43, *P*=.038, η^2^=.046). Those participants whose needs were made salient before the consultation responded to the physician’s communication style, while participants without salient needs did not. When the physician used a need-oriented communication style, those participants with salient needs had a more positive attitude toward mammography after the consultation than before (mean 0.13, SD 0.54), while they changed their attitude in a negative direction when confronted with a purely fact-oriented communication style (mean −0.35, SD 0.80). The same applied to decision modification (need-oriented: mean 0.10, SD 0.99; fact-oriented: mean −0.30, SD 0.88).

**Conclusions:**

The findings underline the importance of communicating in a need-oriented style with patients and advice seekers who are aware of their personal needs. Ignoring the needs of those people appears to be particularly problematic. So physicians’ sensitivity for advice seekers’ currently relevant needs is essential.

## Introduction

### Doctor-Patient Communication on the Internet

People increasingly seek medical consultation and advice on the Internet [1-5]. Many patients and advice seekers are interested in comprehensive online communication with health care professionals [6,7]. The online situation may influence how patients and physicians perceive their roles in the communication process, and this in turn may affect the further development of the doctor-patient relationship [8,9]. The application of purely text-based communication is associated with particular challenges: users cannot easily express themselves in writing and the communication setting provides only reduced social context cues [10]. These characteristics can result in feelings of anonymity [11] and may undermine information exchange [12] and decision making [13], all of which may hinder establishment of interpersonal relationships [14].

With regard to their goals, however, face-to-face and online communication methods have much in common. In both cases, doctor-patient communication serves various purposes: Physicians and advice seekers want to develop a positive interpersonal relationship, exchange information, and come to a point where they can make reasonable medical decisions [15]. This may be facilitated if people feel their needs as advice seekers are recognized and accepted. Positive dialogue should support them in evaluating medical information and making decisions, and a pleasant atmosphere has been shown to have a positive impact on the success of therapy by motivating patients [16-18]. Good doctor-patient communication can improve information exchange, which then leads to informed decisions [17].

Patients and advice seekers come to a medical consultation with various personal needs. It is unclear, however, how their needs and the salience of these needs influence their perception of a doctor’s communication style. Patients’ characteristics, opinions, and needs play an important role in their information processing [19,20]; it is plausible that the interplay of their needs and a physician’s communication style has an impact on their attitudes toward a medical procedure and their related decision making. The aim of the present study was to examine how the salience of advice seekers’ needs in an online consultation and doctors’ communication skills in addressing these needs influence advice seekers’ attitudes toward a medical procedure (mammography screening) and their decisions whether or not to undergo that procedure.

In the following sections, we take the literature on needs of patients and advice seekers into consideration, discuss relevant factors of need-oriented communication, and derive research hypotheses from these considerations. Then we describe the methods of our experimental study and present its results. Concluding, we discuss our findings with respect to their practical implications and provide suggestions for future research.

### Advice Seekers’ Needs

In motivational psychology, needs are relatively stable characteristics that describe the tendency of individuals to pursue particular goals [21]. Patients’ and advice seekers’ needs in consultations can be measured by asking them for their personal hopes and expectations regarding a physician visit or a medical treatment [22]. Surveys have shown that most people have the need to receive clear, balanced, and complete information in a consultation [23-26]. When this need for clarity is addressed during a consultation, patients are more satisfied with the treatment [15].

In addition, people usually try to achieve pleasure and avoid pain [27,28]. Accordingly, in health care situations, patients expect that they will retain or restore their health and well-being through medical treatments [29]. With regard to medical prevention, one key reason why people participate in prevention activities is that they want to stay healthy and feel good [30,31]. Avoiding psychological strain is one important aspect in a woman’s decision about participating in cancer screening [32]. That is, an advice seeker’s need for well-being plays an important role in prevention procedures, such as mammography screening.

Meeting patients’ and advice seekers’ needs is a central challenge for health care [33,34]. It is well known that need fulfillment has many positive consequences. For example, need satisfaction is related to a greater adherence to medical recommendations [35] and to subjective well-being [36].

In a consultation, patients perceive the physician’s communication as need-oriented attention when their needs have been addressed. Even though we may assume that virtually all medical advice seekers possess a need for clarity as well as a need for well-being, we also assume that there are situations where people are more conscious of these needs, that is, where these needs are more or less salient in terms of cognitive accessibility [37]. For example, people who are invited to prepare for a medical consultation [38] or who are explicitly asked about their needs [39] are more conscious of their individual expectations and needs than people who are more indifferent in the medical consultation. The active reflection on one’s needs makes those individual needs more salient. Accordingly, there are situations in health care where (1) advice seekers’ needs are salient and their physicians meet those needs, (2) advice seekers’ needs are salient but their physicians do not meet those needs, and (3) advice seekers’ needs are not salient, making it presumably less relevant whether or not their physicians meet those needs in the communication.

### Physicians’ Need-Oriented Communication

Health communication should be adapted to individual demands and preferences [40,41]. From research on this kind of tailoring and targeting of health information, it is known that if communicated health information meets individual needs, the patients consider the information to be more important [42,43]. In addition, tailored information influences people’s attitudes toward medical procedures such as mammography interventions [44,45] and increases participation in prevention programs [45-47]. The fit between the way health information is communicated and the patient’s or advice seeker’s individual preferences is a critical factor in health communication. Most of the research that investigated tailored health communication focused on personal characteristics such as education and age [48-52] or on clinical features [51]. But patients’ individual needs are also known to influence how information is processed [42,43]. Therefore, it is suggested that the communication style be adapted to the individual needs of a patient or advice seeker [40,41].

When people seek medical advice, they are more or less conscious of their needs, meaning that their needs for clarity and for well-being can be more or less salient. When these needs are salient and people have the impression that their physician takes their needs into account by responding to specific concerns, it may make them more sympathetic to the content of the consultation. So when the fit between their needs and the physician’s communication style affects their information processing correspondingly, these advice seekers would value a medical procedure more highly than when needs and communication style do not fit—if they evaluate the health information positively. This applies to the case of mammography screening, since women have a positive impression of the procedure and even tend to overestimate its benefits [20,53,54]. People also engage more actively in processing information if they perceive the information as personally relevant [55]. This is the case when information is tailored to individual aspects [44,46,47]. In the following study, we investigated whether the salience of advice seekers’ needs and the need-oriented communication style of a physician influence attitudes and decisions about a medical procedure.

### Hypotheses

Hypothesis 1: The salience of an advice seeker’s needs and a physician’s communication style will interact to affect attitude change: People with highly salient needs will have a more positive attitude toward a medical procedure if they encounter a physician who applies a need-oriented communication style instead of a purely fact-oriented communication style. This will not apply to people without salient needs.

Hypothesis 2: The salience of an advice seeker’s needs and a physician’s communication style will interact to affect decision modification: People with highly salient needs will be more willing to undergo a medical procedure if they encounter a physician who applies a need-oriented communication style instead of a purely fact-oriented communication style. This will not apply to people without salient needs.

## Methods

### Study Design and Setting

This study represented a 2 × 2 factorial design with need salience and communication style as between-group factors. The experiment was conducted as an online study where participants took part in a role play of a consultation about mammography screening. Mammography screening is a nationwide, quality-assured breast cancer examination program. In Germany, like in many Western countries, all healthy women aged 50 to 69 years are invited to participate in mammography screening every two years. Younger women are invited if they belong to a high-risk group.

During the consultation, a fictitious, program-controlled physician provided information about advantages and disadvantages of mammography screening. The physician either merely communicated factual medical information or transmitted the same information but made additional comments in a need-oriented communication style by addressing both the need for clarity and the need for well-being. Independently of this encounter, participants’ needs were either measured (and thus made salient) before or after the consultation with a needs questionnaire. This procedure resulted in four experimental conditions: (1) need-oriented communication style/salient needs, (2) fact-oriented communication style/salient needs, (3) need-oriented communication style/no salient needs, (4) fact-oriented communication style/no salient needs.

### Participants

One hundred women aged 20 to 47 years (mean 25.22, SD 4.71) participated in this online role play of a consultation on mammography screening. Women were recruited from volunteers registered in the institutional participant database and invited via email. The database is designed for recruiting study participants. Registration in the database is open to everyone. Four participants were excluded from further analysis because they had already undergone a mammography procedure and thus apparently already made conclusive decisions about mammography screening. This exclusion criterion implied that only women without a breast cancer diagnosis participated in the study. We included only women with German as their native language.

The remaining 96 participants were randomly assigned to one of the four experimental conditions, with 24 participants in the need-oriented communication/salient needs condition, 23 in the fact-oriented communication/salient needs condition, 24 in the need-oriented communication/no salient needs condition, and 25 in the fact-oriented communication/no salient needs condition. The participants in the four experimental conditions did not differ with regard to age (*F*_3,92_=0.82, *P*=.49).

### Procedure and Material

For all participants the experiment started with the same pretest. Included in this pretest were demographic questions as well as measurements of participants’ attitudes toward mammography screening and their hypothetical decision whether or not to participate in a screening (see next section for details on these instruments). Then all participants were introduced to the general experimental situation. Here, they were told to imagine that they had an appointment with their gynecologist in order to gather information about mammography screening. During this appointment they would be able to ask questions that would be answered by the doctor. Participants were told that the physician who answered their questions would not be a real person. They were assured that all answers had been approved as to their medical correctness. They were asked to imagine they were participating in a real doctor-patient dialogue. After that general introduction, participants in the two salient needs conditions filled in a needs questionnaire that made salient their needs for clarity and well-being (see next section for details on this instrument). Then all participants engaged in the interactive online role play that took place as a text chat between the participants and a fictitious, program-controlled physician. In this role play the physician provided information about advantages and disadvantages of mammography screening. The physician either merely communicated factual information about mammography screening (in the two fact-oriented communication style conditions) or made additional comments using a need-oriented communication style (in the two need-oriented communication style conditions) where he explicitly addressed the needs for clarity and well-being.

In the online role play the fictitious physician provided participants in all four conditions with the same two advantages and two disadvantages of mammography screening. The sequence in which the participants encountered these information items in the text chat differed, however, depending on their replies to the posts of the physician character. The potential sequences of interactions in the online role play are presented in Multimedia Appendix 1. For each step of communication, the participants could choose their question or answer from a predetermined selection of text modules. The role play started with a participant’s opening question to which the physician either replied in a need-oriented or a purely fact-oriented manner. In the need-oriented communication style conditions, the physician emphasized, for example, that he understood that this conversation was about obtaining clarity on what exactly mammography screening is about or that well-being was important for this participant. These statements demonstrated to the participants that the doctor had recognized their needs and was willing to consider them explicitly. In the fact-oriented communication style conditions, the physician refrained from emphasizing those needs for clarity and for well-being.

The role play was technically implemented as an online questionnaire using the Enterprise Feedback Suite (Questback) as an online survey system [56]. During the role play participants were able to interact with the virtual physician by choosing one of several possible statements in each trial. With respect to their chosen answer, they were then dynamically forwarded to the next site of the role play that presented the next piece of information provided by the physician. To ensure that all four conditions of the role play presented the same information to the participants, the conversation parts that presented information about the mammography screening were identical in their wording in the different conditions. The need-oriented comments were separate and over and above these factual statements.

Following the role play, all participants filled in the same posttest questionnaire that again measured their attitude toward mammography screening and their hypothetical decision whether or not to participate. These measurements were identical to those in the pretest. In addition, the posttest asked participants to assess the arguments about mammography screening given to them during the role play to ensure that they valued the advantages of mammography screening as presented by the physician. Moreover, they replied to the item *I was easily able to put myself in the consultation situation* (immersion item) on a 5-point scale (1=*I do not agree* to 5=*I totally agree*). Finally, participants in the two no salient needs conditions filled in the needs questionnaire.

### Instruments

As pointed out above, we measured all participants’ attitudes toward mammography screening and their hypothetical decisions whether or not to participate before and after the experiment. The attitude test consisted of four pairs of adjectives which participants had to rate on 7-point semantic differential scales (see Table 1). This text was based on the attitude measurement by Marteau, Dormandy, and Michie [57].

The decision measurement consisted of two items that participants had to rate on 5-point Likert scales ranging from 1=*does not apply at all* to 5=*applies completely* (see Table 2).

**Table 1:** Attitude scale.

I think that for me participation in mammography screening at the age of 50 is…	
1	advantageous □ □ □ □ □ □ □ disadvantageous^r^
2	important □ □ □ □ □ □ □ unimportant^r^
3	a bad thing □ □ □ □ □ □ □ a good thing
4	convenient □ □ □ □ □ □ □ inconvenient^r^

^r^Indicates reversely coded items.

**Table 2:** Decision scale.

1	I consider participation in mammography screening to be very reasonable.
2	I would participate in breast cancer screening using mammography.

Participants’ needs for clarity and for well-being were measured with a needs questionnaire. These scales were developed on the basis of the literature mentioned above [22-32] and designed to capture the broadness of the constructs. Hence, the items of the need for clarity scale captured how important it is for advice seekers to receive instructive and useful information in a consultation about mammography screening. The items of the need for well-being scale inquired about how important it is for advice seekers to stay healthy and maintain their status of well-being. Each scale consisted of seven items that participants had to rate on 5-point Likert scales ranging from 1=*does not apply at all* to 5=*applies completely*. The need for clarity scale is presented in Table 3, the need for well-being scale in Table 4.

Participants assessed the arguments (advantages and disadvantages) on mammography screening that were given to them during the role play on 7-point Likert scales ranging from 1=*very unimportant* to 7=*very important* (see Table 5).

**Table 3:** Need for clarity scale.

1	For me it is important to receive very structured counseling on mammography screening.
2	For me it is important to understand what happens to me during a mammography examination.
3	For me it is important to understand the meaning of the findings of mammography screening.
4	For me it is important to be told comprehensively about the advantages and risks of mammography screening.
5	For me it is important to comprehend what benefits I get from mammography screening.
6	For me it is important to comprehend what the screening cannot achieve.
7	For me it is important to be informed why mammography screening could be more or less reasonable for me.

**Table 4:** Need for well-being scale.

1	For me it is important to be sure that I am really healthy.
2	I would do anything to stay healthy.
3	For me it is important not to expose myself to health risks.
4	For me it is important to be psychologically and physically well.
5	For me it is important to do anything to reduce the risk of dying of breast cancer.
6	I do not want to expose myself to psychological strain.
7	I do not want to worry for no reason.

**Table 5:** Assessment of arguments about mammography screening.

1	Through early detection of a malignant tumor, it can be treated more mildly, the breast can be preserved, for example, and one can refrain from chemotherapy.^a^
2	A diagnostic finding may turn out to be without cause, causing tissue to be removed that later proves to be benign.^b^
3	A malignant tumor that would have been lethal without examination can be detected in a curable stage.^a^
4	A malignant tumor might be detected and treated that is not curable anymore, which would not prolong life but may prolong suffering.^d^

^a^Indicates an argument about advantages.

^b^Indicates an argument about disadvantages.

### Statistical Analysis

We compared the empirical values of the immersion item against the scale midpoint using a one-sample *t* test. In order to assess their internal consistencies, we calculated Cronbach alpha values of all of the scales. A basic precondition for validly testing the two hypotheses was that the needs for clarity and for well-being were actually relevant needs for our participants. In order to test the fulfilment of this precondition, we compared the empirical values against the scale midpoints using one-sample *t* tests. We compared participants’ needs among the four conditions with analyses of variance (ANOVAs). To examine participants’ assessment of advantages and disadvantages, we calculated paired samples *t* tests. For both attitude and decision we tested whether there were changes from the pretest to the posttest in the overall sample, applying paired samples *t* tests.

In order to test Hypothesis 1, which predicted an interaction effect of need salience and communication style on attitude change, we calculated the difference between participants’ attitudes in the pretest and the posttest. Accordingly, a negatively signed value indicates a more negative attitude after the online role play than before the consultation, and a positively signed value indicates a more positive attitude than before the consultation. In order to test Hypothesis 2, which predicted an interaction effect of need salience and communication style on decision modification, we calculated the difference between participants’ decisions in the pretest and the posttest. Hence, a negatively signed value indicates a stronger tendency not to participate in mammography screening after the online role play than before, and a positively signed value indicates a stronger tendency to participate after the consultation. To test Hypotheses 1 and 2 we used ANOVAs. In order to compare individual conditions we applied least-significant-difference tests as post hoc tests.

### Ethical Considerations

This study had full ethical approval of the Leibniz-Institut fuer Wissensmedien ethics committee (approval number: LEK 2013/043). Participants were informed about privacy protection and their right to terminate participation at any time without any disadvantage. They participated voluntarily and anonymously. They were debriefed at the end of the experiment.

## Results

### Immersion

As a first step we analyzed whether the participants stated that they were able to put themselves in the fictitious consultation situation. Their rating (mean 3.74, SD 1.17) was significantly higher than the midpoint (3) of the 5-point scale: *t*_94_=6.14, *P*<.001, *d*=0.63. This indicates that the participants were able to immerse mentally into the situation.

### Needs

The need for clarity scale had an internal consistency of α=.74. The need for well-being scale had an internal consistency of α=.76. The participants expressed strong needs for clarity (mean 4.57, SD 0.42) and for well-being (mean 4.21, SD 0.54) with both means being significantly higher than the midpoint (3) of the 5-point Likert scale (need for clarity: *t*_95_=36.68, *P*<.001, *d*=3.74; need for well-being: *t*_95_=21.97, *P*<.001, *d*=2.24). This finding strongly indicates that both needs of which we intended to make participants aware were real needs for them. The needs did not differ among the participants in the four experimental conditions, neither regarding the need for clarity (*F*_*3,92*_=0.75, *P*=.53) nor regarding the need for well-being (*F*_*3,92*_=0.76, *P*=.52).

### Assessment of Arguments

The items on advantages and disadvantages were summarized in two subscales. The advantages subscale had a good internal consistency of α=.70. The disadvantages subscale, however, had an unacceptable internal consistency of α=.21. Apparently, the two disadvantages represented quite different types of reasons for the participants. Thus, we considered the disadvantages items separately but the advantages as a scale: The participants assessed the advantages (mean 6.63, SD 0.70) to be much more important than the first disadvantage item (*tissue removal without cause*; mean 4.03, SD 1.62; *t*_94_=15.55, *P*<.001, *d*_*z*_=1.59) and more important than the second disadvantage item (*prolonged suffering*; mean 4.35, SD 1.67; *t*_95_=12.28, *P*<.001, *d*_*z*_=1.26).

### Attitude Change

The attitude scale had an internal consistency of α=.75 in the pretest and α=.74 in the posttest. Across all four conditions, the participants’ attitudes did not differ between the pretest (mean 5.60, SD 0.86) and the posttest (mean 5.50, SD 0.96) (*t*_95_=1.69, *P*=.094). However, both mean values differed significantly from the midpoint (4) of the 7-point scale (with higher values representing a more positive attitude): pretest (*t*_95_=18.17, *P*<.001, *d*=1.86) and posttest (*t*_95_=15.35, *P*<.001, *d*=1.56), indicating an overall positive attitude toward mammography screening.

**Figure 1:**
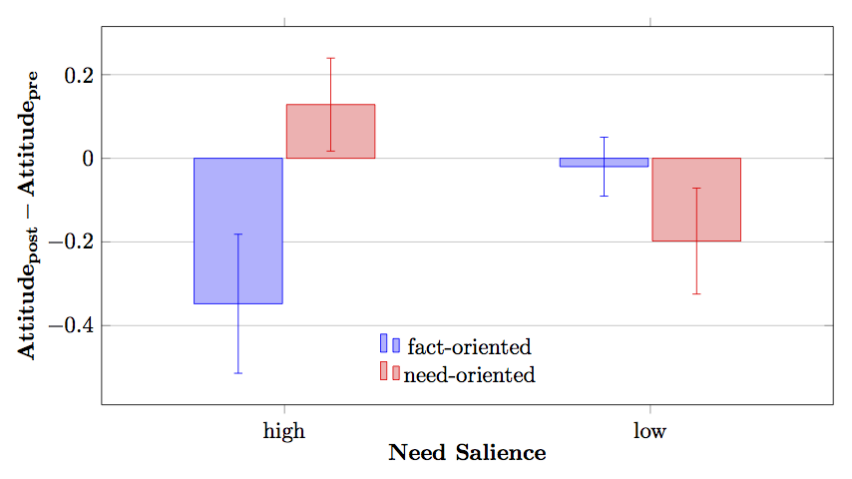
Interaction effect between communication style and need salience regarding attitude change. Standard errors are represented by error bars attached to each column.

As assumed in Hypothesis 1, we found a significant interaction effect of need salience and communication style on attitude change (*F*_*1,92*_=7.23, *P*=.009, η^2^=.073). Those participants whose needs were salient responded to the physician’s communication style, while participants without salient needs did not (see Figure 1). In the case of need-oriented communication by the physician character, those participants with salient needs showed an attitude change in a positive direction (mean 0.13, SD 0.54), while they changed their attitude in a negative direction when confronted with a purely fact-oriented communication style (mean −0.35, SD 0.80). With salient needs, communication style had a differential effect on attitude change (*P*=.007), which did not occur for people without salient needs (*P*=.299).

### Decision Modification

The decision scale had an internal consistency of α=.96 in the pretest and α=.94 in the posttest. Across all four conditions, the participants’ decision did not differ between the pretest (mean 4.42, SD 0.94) and the posttest (mean 4.37, SD 0.92) (*t*_95_=0.69, *P*=.495). However, both mean values differed significantly from the midpoint (3) of the 5-point scale (with higher values representing a stronger tendency to participate in mammography screening): pretest (*t*_95_=14.85, *P*<.001, *d*=1.51) and posttest (*t*_95_=14.66, *P*<.001, *d*=1.49), indicating an overall strong tendency to participate in mammography screening.

**Figure 2:**
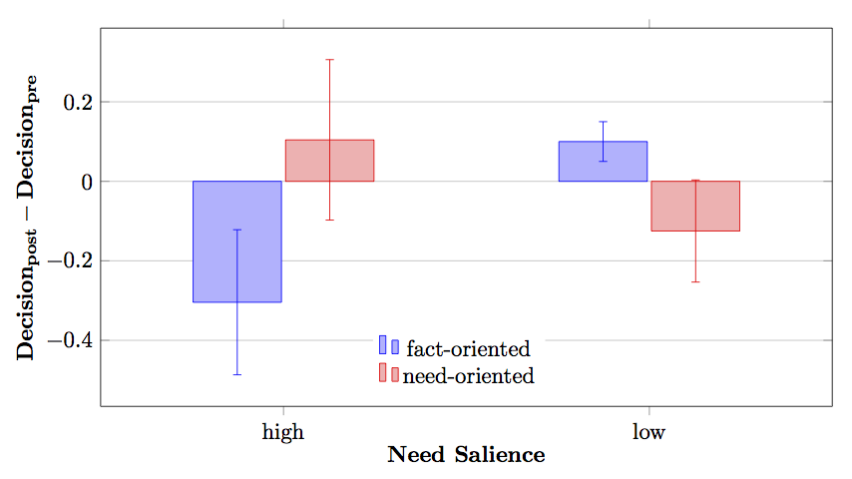
Interaction effect between communication style and need salience regarding decision modification. Standard errors are represented by error bars attached to each column.

As assumed in Hypothesis 2, we found a significant interaction effect of need salience and communication style on decision modification (*F*_*1,92*_=4.43, *P*=.038, η^2^=.046). Participants with salient needs responded to the physician’s communication style in the consultation, whereas participants without salient needs did not (see Figure 2). When the physician character applied a need-oriented communication style, those participants with salient needs were more willing to participate in mammography screening after the consultation than before (mean 0.10, SD 0.99), while they were less willing to participate given a fact-oriented communication style (mean −0.30, SD 0.88). With salient needs, communication style tended to have a differential effect on decision modification (*P*=.06), which we did not find for people without salient needs (*P*=.29).

## Discussion

### Principal Findings

As expected, our participants expressed strong needs for clarity and for well-being. Participants had an overall positive attitude toward mammography screening and a strong tendency to be willing to participate in that procedure. We provided them with balanced information regarding advantages and disadvantages of mammography screening during the role play, but we found that they assessed advantages of mammography screening to be more important than disadvantages. These findings are in line with other research findings showing that women often overestimate the advantages of mammography screening [53,54].

The main goal of the study was to investigate to what extent patients’ attitudes toward mammography screening and their decisions whether or not to participate in that procedure were influenced by the interplay between the salience of patients’ needs and a physician’s ability to be more or less responsive to these needs in an online consultation. Regarding attitude change and decision modification, we found that those participants whose needs were salient in the consultation responded to the physician’s communication style, while participants without salient needs did not. With a need-oriented communication style, those participants whose needs for clarity and well-being had been made salient showed an attitude change in a positive direction (corresponding to their high valuation of the advantages of mammography screening), while they changed their attitude in a negative direction when given a purely fact-oriented communication style. The same pattern of development applied to decision modification. These results are even more remarkable since the information provided in the online role play was balanced, and only one tailoring strategy was used (a meta-analytic review [46] pointed out that tailoring using several different strategies tends to be more effective than using just one strategy). It seems that women were more sympathetic to mammography screening and its advantages regardless of the physician character’s effort to provide both advantages and disadvantages of this procedure. It appears that this positive evaluation of the procedure rubbed off on the modification of their attitudes and decisions when their needs were addressed by the physician—otherwise they developed in the opposite direction.

### Limitations and Future Work

A limitation of this study is that generalization of the findings to the whole population of women and to real (online) consultations must be handled with care. We cannot be certain to what extent women would decide in the same way if they were really faced with the decision whether or not to take part in mammography screening. In addition, we cannot know from the current findings to what extent women would react the same way if they were not confronted with a purely text-based consultation but with a richer [58] online communication, allowing the transfer of more social context cues [10]. It would also be interesting to test a similar setting in a face-to-face situation, in particular since there is evidence that tailored health messages are also an effective approach in face-to-face communication [59].

Another limitation is that we focused only on the needs for clarity and for well-being. It is possible that taking other personal needs into account would yield quite different results. In future studies it would be interesting to compare attitude change and decision modification of women of differing ages and to consider other personal needs that might be relevant to medical consultation and decision-making. In addition, it would be worthwhile to take people’s knowledge acquisition into account as well, in order to test whether their decision is an informed decision based on appropriate knowledge about the risks and benefits of a medical treatment.

The automatic response system that we developed for the study reported here might not have been entirely adequate as a representation of a real online consultation. Perhaps the participants experienced the communication as artificial or felt constricted in their choices to express their concerns and requests. However, participants were able to put themselves properly into the situation. Even so, the informative value of this one-item measurement needs to be handled with care. It would be interesting to replicate this study with real synchronous online communication. This might even increase the effects reported in this article.

### Conclusions

The interaction effects of patients’ need salience and physicians’ communication style yield remarkable results, because they point out the importance of communicating in a need-oriented style with patients or advice seekers who are mindful of their personal needs in a given situation. When their personal needs were made salient, it seemed to be especially important to participants to have these needs met. Apparently, the needs for clarity and for well-being were not necessarily consciously accessible and were only relevant in an online consultation situation when they were activated (ie, made cognitively accessible) in advance.

So we conclude that physicians’ sensitivity to their patients’ currently relevant needs is essential. This is true not only because need-oriented communication resulted for people with highly salient needs in a more positive attitude toward the content of the consultation and a higher willingness to participate, but also because ignoring the needs of those people had the opposite effects. Communication style had a particularly strong effect when needs were currently relevant but were then disregarded by the physicians. In a counselling situation, one way for a physician to become aware of the needs of patients or advice seekers is to ask them for their expectations about the consultation and a medical intervention that might potentially result. The very same approach would also raise the patients’ or advice seekers’ awareness of their own needs and would make these needs more salient accordingly. In this way, it is possible for health care professionals to recognize which needs are currently relevant for a patient and to address these needs in their communication.

## Appendix

Multimedia Appendix 1 Sequences of interactions in the online role play.
