# Supplementary figures and images for: The Impact of Advice Seekers’ Need Salience and Doctors’ Communication Style on Attitude and Decision Making: A Web-Based Mammography Consultation Role Play
Source: JMIR Cancer. 2015 Sep 8;1(2):e10. doi: 10.2196/cancer.4279 (PMC5367665; doi:10.2196/cancer.4279)

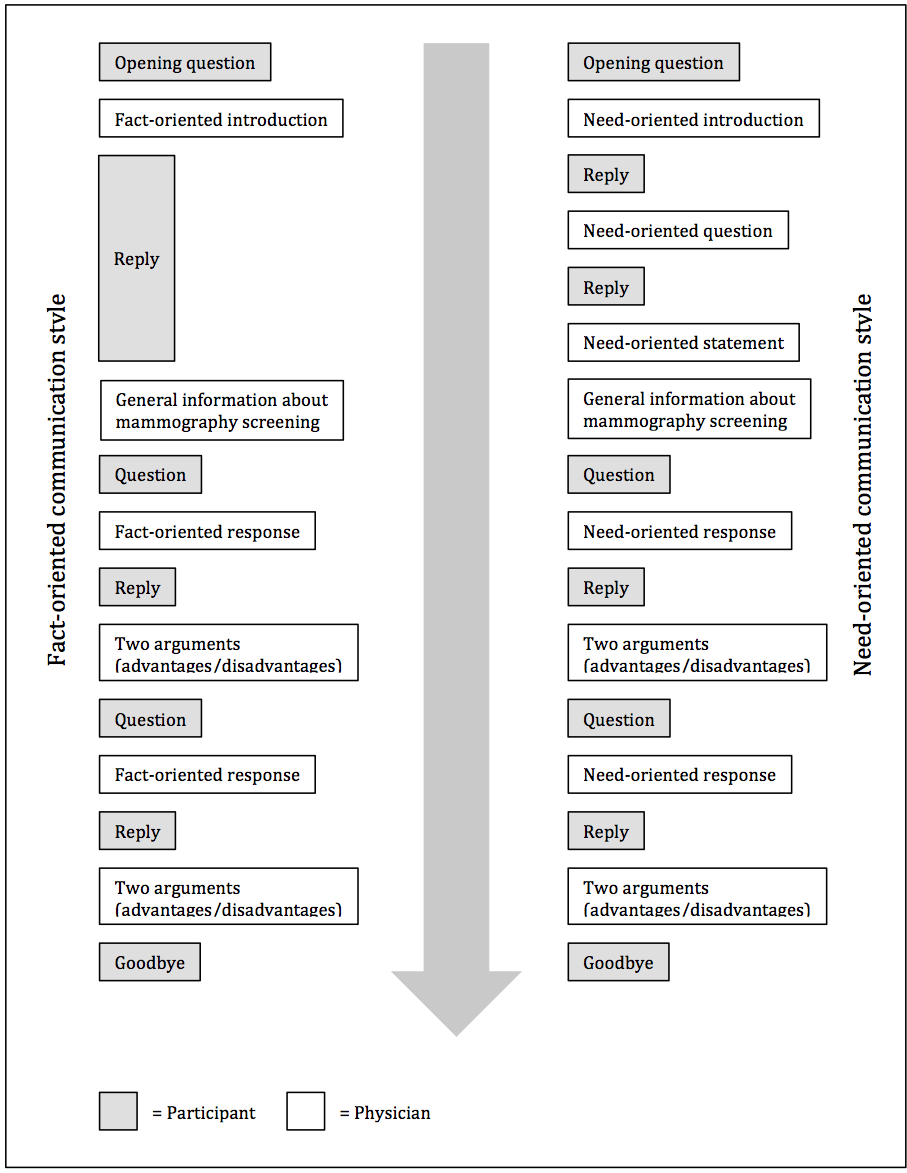

Supplement: Multimedia Appendix 1 [file cancer_v1i2e10_app1.png]
